# Supplementary material for: Serum Adipokines, Growth Factors, and Cytokines Are Independently Associated with Stunting in Bangladeshi Children
Source: Nutrients. 2019 Aug 7;11(8):1827. doi: 10.3390/nu11081827 (PMC6723106; doi:10.3390/nu11081827)
Supplement: Supplementary file 1 [file nutrients-11-01827-s001.pdf]

Supplementary File

Table S1: Spearman correlation between different biomarkers at enrolment in stunted children

At enrolment	AAT	MPO	NEO	Ghrelin	Leptin	Adiponectin	OXM	GLP-1	GLP-2	IGF-1	FGF-19	FGF-21	IL-1B	IL-4	IL-6	IL-10	IL-12	TNF	IFN-γ	CRP
**AAT**	1.00																			
**MPO**	0.16	1.00																		
**NEO**	0.12	-0.02	1.00																	
**Ghrelin**	0.14	0.24	-0.09	1.00																
**Leptin**	0.23	-0.08	-0.08	0.08	1.00															
**Adiponectin**	0.24	0.04	0.02	0.09	0.27	1.00														
**OXM**	0.14	-0.01	0.20	0.16	-0.08	0.05	1.00													
**GLP-1**	-0.01	-0.01	0.22	0.04	0.12	0.02	0.39	1.00												
**GLP-2**	0.00	0.34	0.15	0.00	-0.18	-0.05	0.17	-0.02	1.00											
**IGF-1**	0.12	-0.04	0.16	-0.01	0.34	0.01	0.15	0.33	0.01	1.00										
**FGF-19**	-0.02	0.11	-0.25	0.07	0.10	-0.04	-0.04	-0.10	-0.11	-0.13	1.00									
**FGF-21**	-0.04	0.04	-0.06	-0.06	-0.25	-0.01	0.11	0.06	0.19	-0.20	0.08	1.00								
**IL-1B**	0.23	0.08	0.12	0.12	0.02	0.31	-0.03	0.08	-0.29	0.02	-0.04	-0.26	1.00							
**IL-4**	0.24	0.05	0.38	0.10	0.04	0.18	0.41	0.39	0.01	0.25	-0.28	0.04	0.17	1.00						
**IL-6**	0.14	0.03	0.03	0.22	-0.12	0.17	0.48	0.44	0.07	0.34	-0.15	-0.04	0.12	0.49	1.00					
**IL-10**	0.23	-0.14	0.01	0.20	-0.10	0.08	0.15	0.08	0.11	0.06	-0.09	0.32	-0.02	0.45	0.32	1.00				
**IL-12**	0.16	0.07	0.35	0.23	0.00	0.04	0.43	0.53*	0.12	0.39	-0.20	-0.10	0.20	0.59*	0.52*	0.16	1.00			
**TNF**	0.16	-0.14	0.07	0.23	-0.17	-0.13	-0.11	-0.28	0.28	-0.11	-0.09	0.22	-0.22	0.12	0.06	0.62*	0.01	1.00		
**IFN-γ**	-0.01	-0.25	-0.06	0.00	-0.31	0.07	0.02	0.17	0.00	-0.21	-0.26	0.22	0.02	0.01	0.16	0.36	-0.03	0.27	1.00	
**CRP**	-0.07	0.13	0.04	0.11	-0.30	0.01	0.11	0.05	0.09	-0.16	-0.08	-0.24	0.13	0.34	0.46	0.30	0.08	0.18	0.13	1.00

Data are analyzed by Spearman's rank correlation coefficient or Spearman's rho or ϱ: *p <0.05. All estimates are sera based. Abbreviation: AAT: alpha-1 antitrypsin; CRP: C-reactive protein; FGF: fibroblast growth factor; IFN-γ: gamma interferon; GLP: glucagon-like peptide; IL: interleukins; IGF: insulin-like growth factor; MPO: myeloperoxidase; NEO: neopterin; OXM: oxyntomodulin; TNF-α: tumor necrosis factor alpha.

Table S2: Spearman correlation between different biomarkers at 6-month post-enrolment in stunted children

At 6-month post-enrolment	AAT	MPO	NEO	Ghrelin	Leptin	Adiponectin	OXM	GLP-1	GLP-2	IGF-1	FGF-19	FGF-21	IL-1B	IL-4	IL-6	IL-10	IL-12	TNF	IFN-γ	CRP
**AAT**	1.00																			
**MPO**	-0.09	1.00																		
**NEO**	0.10	-0.06	1.00																	
**Ghrelin**	0.17	0.09	0.02	1.00																
**Leptin**	-0.13	0.06	-0.25	-0.01	1.00															
**Adiponectin**	-0.08	0.15	0.13	0.01	0.26	1.00														
**OXM**	-0.04	0.11	-0.01	0.20	0.12	-0.25	1.00													
**GLP-1**	0.18	-0.29	0.55	-0.17	-0.19	0.14	-0.10	1.00												
**GLP-2**	0.02	0.07	0.22	-0.33	0.17	0.08	-0.09	0.18	1.00											
**IGF-1**	-0.10	-0.38	-0.13	0.12	0.30	0.11	0.09	0.10	-0.12	1.00										
**FGF-19**	0.31	0.21	-0.05	-0.04	-0.04	0.10	0.05	0.10	-0.05	-0.28	1.00									
**FGF-21**	0.20	-0.10	-0.24	0.17	-0.12	-0.41	-0.02	-0.23	-0.08	-0.23	0.24	1.00								
**IL-1B**	-0.05	0.02	0.02	0.23	0.13	0.09	0.32	-0.12	-0.19	0.00	-0.22	0.20	1.00							
**IL-4**	0.13	-0.13	-0.07	0.17	-0.09	-0.05	0.02	0.01	-0.20	-0.02	0.13	0.32	-0.09	1.00						
**IL-6**	0.00	-0.01	-0.09	0.17	-0.01	0.01	-0.04	-0.11	0.08	-0.07	-0.15	0.14	0.22	0.14	1.00					
**IL-10**	0.14	0.30	0.12	-0.14	-0.25	-0.25	-0.08	0.17	0.22	-0.38	-0.04	0.03	-0.08	0.02	0.42	1.00				
**IL-12**	-0.46	0.20	0.05	-0.04	0.30	0.10	-0.12	0.13	0.15	0.29	-0.54	-0.29	0.18	-0.18	0.06	0.14	1.00			
**TNF**	-0.04	0.27	0.05	-0.09	-0.05	0.04	-0.06	-0.02	0.19	0.06	-0.20	0.06	0.12	0.07	0.10	0.17	0.25	1.00		
**IFN-γ**	0.00	0.18	0.06	0.08	0.02	0.00	-0.03	0.05	-0.02	-0.06	-0.35	-0.07	0.06	-0.13	0.20	0.30	0.41	0.32	1.00	
**CRP**	0.04	0.15	-0.16	0.30	-0.24	-0.17	0.12	-0.11	-0.17	-0.24	-0.06	0.24	0.20	0.24	0.58*	0.35	-0.05	0.03	-0.11	1.00

Data are analyzed by Spearman's rank correlation coefficient or Spearman's rho or ϱ: *p <0.05. All estimates are sera based. Abbreviation: AAT: alpha-1 antitrypsin; CRP: C-reactive protein; FGF: fibroblast growth factor; IFN-γ: gamma interferon; GLP: glucagon-like peptide; IL: interleukins; IGF: insulin-like growth factor; MPO: myeloperoxidase; NEO: neopterin; OXM: oxyntomodulin; TNF-α: tumor necrosis factor alpha.

Table S3: Spearman correlation between different biomarkers at enrolment in control children

At enrolment	AAT	MPO	NEO	Ghrelin	Leptin	Adiponectin	OXM	GLP-1	GLP-2	IGF-1	FGF-19	FGF-21	IL-1B	IL-4	IL-6	IL-10	IL-12	TNF	IFN-γ	CRP
**AAT**	1.00																			
**MPO**	0.12	1.00																		
**NEO**	-0.09	0.19	1.00																	
**Ghrelin**	-0.20	0.07	0.34	1.00																
**Leptin**	0.11	-0.30	-0.12	-0.06	1.00															
**Adiponectin**	-0.42	-0.26	-0.08	0.03	0.08	1.00														
**OXM**	0.31	-0.13	-0.08	-0.02	0.83*	-0.44	1.00													
**GLP-1**	-0.12	0.29	0.00	0.22	0.01	0.04	0.01	1.00												
**GLP-2**	0.01	-0.19	0.31	0.34	0.00	-0.03	0.08	0.16	1.00											
**IGF-1**	-0.09	0.07	-0.05	-0.12	-0.20	0.38	-0.36	0.23	0.03	1.00										
**FGF-19**	-0.05	-0.11	0.11	0.02	0.55*	0.02	0.48	0.05	0.11	-0.02	1.00									
**FGF-21**	-0.23	0.28	0.16	0.01	-0.43	0.01	-0.39	-0.12	-0.06	-0.17	-0.17	1.00								
**IL-1B**	0.15	0.21	-0.11	0.07	0.04	0.01	0.03	0.13	0.06	-0.21	-0.22	-0.03	1.00							
**IL-4**	-0.02	0.01	0.16	0.18	-0.06	-0.11	0.04	-0.01	0.13	-0.42	-0.02	0.19	0.32	1.00						
**IL-6**	-0.03	-0.07	0.04	0.10	0.00	0.08	0.00	0.27	0.23	-0.14	0.06	0.08	0.00	0.14	1.00					
**IL-10**	-0.03	0.10	0.08	0.32	-0.10	-0.11	0.01	0.11	0.16	0.19	0.04	-0.02	-0.16	-0.01	0.00	1.00				
**IL-12**	0.13	0.29	0.19	0.10	-0.10	-0.02	-0.09	0.22	0.18	0.18	-0.07	0.00	0.03	0.13	0.11	0.30	1.00			
**TNF**	-0.09	0.00	0.14	0.09	-0.11	-0.15	-0.04	0.19	0.27	-0.01	0.16	0.11	-0.08	0.06	0.23	-0.10	0.09	1.00		
**IFN-γ**	0.11	0.24	0.13	0.22	0.06	-0.02	0.10	0.40	0.11	0.12	-0.02	-0.05	0.31	0.20	0.13	0.02	0.42	-0.15	1.00	
**CRP**	-0.02	0.04	0.36	0.11	-0.27	0.17	-0.39	0.11	-0.06	0.08	-0.31	0.15	0.00	0.11	0.18	-0.10	0.34	0.00	0.16	1.00

Data are analyzed by Spearman's rank correlation coefficient or Spearman's rho or ϱ: *p <0.05. All estimates are sera based. Abbreviation: AAT: alpha-1 antitrypsin; CRP: C-reactive protein; FGF: fibroblast growth factor; IFN-γ: gamma interferon; GLP: glucagon-like peptide; IL: interleukins; IGF: insulin-like growth factor; MPO: myeloperoxidase; NEO: neopterin; OXM: oxyntomodulin; TNF-α: tumor necrosis factor alpha.

Table S4: Spearman correlation between different biomarkers at 6-month post-enrolment in control children

At 6-month post-enrolment	AAT	MPO	NEO	Ghrelin	Leptin	Adiponectin	OXM	GLP-1	GLP-2	IGF-1	FGF-19	FGF-21	IL-1B	IL-4	IL-6	IL-10	IL-12	TNF	IFN-γ	CRP
**AAT**	1.00																			
**MPO**	0.02	1.00																		
**NEO**	0.24	0.16	1.00																	
**Ghrelin**	0.29	0.24	0.39	1.00																
**Leptin**	-0.11	0.13	0.20	0.23	1.00															
**Adiponectin**	0.01	-0.12	-0.16	0.17	-0.25	1.00														
**OXM**	-0.15	0.17	0.09	-0.02	0.81*	-0.65	1.00													
**GLP-1**	0.56	0.17	0.29	0.07	0.19	0.09	0.08	1.00												
**GLP-2**	0.09	0.22	-0.10	-0.22	0.11	-0.33	0.39	0.29	1.00											
**IGF-1**	0.04	-0.08	0.27	0.28	0.07	0.30	-0.20	0.18	-0.12	1.00										
**FGF-19**	-0.22	-0.18	-0.12	0.19	0.05	0.18	-0.12	-0.07	-0.26	0.07	1.00									
**FGF-21**	-0.07	0.02	-0.24	-0.52	-0.17	-0.36	0.07	-0.34	0.27	-0.31	-0.04	1.00								
**IL-1B**	-0.05	0.11	0.18	-0.27	0.23	-0.20	0.34	-0.13	-0.03	-0.66	-0.24	0.15	1.00							
**IL-4**	-0.17	-0.40	0.11	-0.22	0.00	0.35	-0.27	-0.12	-0.10	0.21	0.12	0.34	0.01	1.00						
**IL-6**	0.07	-0.04	0.18	-0.47	-0.32	-0.14	-0.19	0.19	-0.14	-0.16	-0.06	0.35	0.15	0.19	1.00					
**IL-10**	0.35	0.08	0.13	0.35	0.00	-0.19	-0.04	0.06	0.02	0.28	0.03	-0.09	-0.26	-0.13	-0.27	1.00				
**IL-12**	-0.09	0.07	0.45	0.24	0.22	-0.14	0.06	0.08	0.01	0.16	0.14	-0.08	0.12	0.23	-0.20	0.41	1.00			
**TNF**	-0.42	-0.11	0.11	-0.25	0.22	-0.10	0.22	-0.02	0.17	0.22	0.00	0.21	-0.02	0.48	0.15	0.07	0.41	1.00		
**IFN-γ**	-0.26	0.26	-0.08	0.03	0.06	0.48	-0.19	0.09	-0.09	0.48	-0.02	0.01	-0.29	0.43	0.00	-0.06	0.16	0.42	1.00	
**CRP**	-0.08	0.10	0.27	-0.01	-0.31	0.23	-0.49	-0.15	-0.10	0.23	0.13	0.19	-0.02	0.41	0.29	0.20	0.47	0.15	0.30	1.00

Data are analyzed by Spearman's rank correlation coefficient or Spearman's rho or ϱ: *p <0.05. All estimates are sera based. Abbreviation: AAT: alpha-1 antitrypsin; CRP: C-reactive protein; FGF: fibroblast growth factor; IFN-γ: gamma interferon; GLP: glucagon-like peptide; IL: interleukins; IGF: insulin-like growth factor; MPO: myeloperoxidase; NEO: neopterin; OXM: oxyntomodulin; TNF-α: tumor necrosis factor alpha.
